# O6-methylguanine-DNA methyltransferase modulates cisplatin-induced DNA double-strand breaks by targeting the homologous recombination pathway in nasopharyngeal carcinoma

**DOI:** 10.1186/s12929-020-00699-y

**Published:** 2021-01-04

**Authors:** Shang-Hung Chen, Wen-Tsung Huang, Wan-Chen Kao, Sheng-Yen Hsiao, Hsin-Yi Pan, Chin-Wen Fang, Yow-Ling Shiue, Chia-Lin Chou, Chien-Feng Li

**Affiliations:** 1 National Institute of Cancer Research, National Health Research Institutes, Tainan, Taiwan; 2 Department of Oncology, National Cheng Kung University Hospital, College of Medicine, National Cheng Kung University, Tainan, Taiwan; 3 Division of Hematology-Oncology, Department of Internal Medicine, Chi Mei Medical Center, Liouying, Tainan, Taiwan; 4 Institute of Clinical Medicine, College of Medicine, National Cheng Kung University, Tainan, Taiwan; 5 Institute of Biomedical Sciences, National Sun Yat-Sen University, Kaohsiung, Taiwan; 6 Division of Colon and Rectal Surgery, Department of Surgery, Chi Mei Medical Center, No. 901, Zhonghua Rd., Yongkang Dist., Tainan, 71004 Taiwan; 7 Department of Medical Research, Chi Mei Medical Center, Tainan, Taiwan; 8 Institute of Precision Medicine, National Sun Yat-sen University, No.70, Lien-hai Rd., Kaohsiung, 80424 Taiwan

**Keywords:** MGMT, Cisplatin, Homologous recombination, PARP inhibitor, Nasopharyngeal carcinoma

## Abstract

**Background:**

The homologous recombination (HR) pathway is involved in DNA damage response (DDR), which is crucial to cancer cell survival after treatment with DNA damage agents. O6-methylguanine DNA methyltransferase (MGMT) is associated with cisplatin (CDDP) resistance in cancer cells; however, the underlying mechanisms remain unclear. Here, we explored the interactions between MGMT and the HR pathway in CDDP-activated DDR and their clinical implications in nasopharyngeal carcinoma (NPC).

**Methods:**

Human NPC cells were assessed using loss-of-function approaches in vitro. The expression correlations between MGMT and major proteins of the HR pathway were analyzed through Western blotting, quantitative real-time PCR, and bioinformatic analysis by using a public database. The physical interactions between MGMT and HR proteins were studied using co-immunoprecipitation and immunofluorescence analyses. Cell comet tails and γ-H2AX expression levels were examined to evaluate double-strand break (DSB) formation. Established immunofluorescence and reporter analyses were conducted to measure HR activity. Xenograft and cell viability studies were used to assess the therapeutic potential of MGMT inhibition in combination with CDDP and poly(ADP-ribose) polymerase (PARP) inhibitor, respectively.

**Results:**

Among major proteins of the HR pathway, MGMT suppression inhibited CDDP-induced *RAD51* expression. Bioinformatic analyses showed a positive correlation between *MGMT* and *RAD51* expression in patients with NPC. Moreover, MGMT physically interacted with BRCA1 and regulated CDDP-induced BRCA1 phosphorylation (ser 988). In functional assays, MGMT inhibition increased CDDP-induced DSB formation through attenuation of HR activity. NPC xenograft studies demonstrated that MGMT inhibition combined with CDDP treatment reduced tumor size and downregulated RAD51 expression and BRCA1 phosphorylation. Furthermore, MGMT suppression increased PARP inhibitor–induced cell death and DSB formation in NPC cells.

**Conclusion:**

MGMT is crucial in the activation of the HR pathway and regulates DDR in NPC cells treated with CDDP and PARP inhibitor. Thus, MGMT is a promising therapeutic target for cancer treatments involving HR-associated DDR.

## Background

Nasopharyngeal carcinoma (NPC), a group of malignant diseases categorized into head and neck cancers, is considered endemic in Southeast Asian countries, including Taiwan [1, 2]. Owing to anatomic characteristics different from those of other head and neck cancers, the main treatment strategy for locally advanced NPC is cisplatin (CDDP)-based chemoradiotherapy (CRT). Several randomized clinical trials have demonstrated that CDDP-based CRT significantly improved the overall survival of patients with advanced-stage NPC [3–5]. Moreover, because of the high response rates in tumor control, CDDP-based chemotherapy regimens are recommended as the first-line treatment in patients with metastatic NPC [6, 7]. Therefore, methods of overcoming CDDP resistance in patients with NPC requires further exploration.

CDDP, a platinum-based anticancer agent, is effective against several types of human cancers [8, 9]. In general, CDDP can induce apoptotic cell death through platinum–DNA adduct formation. Given that this type of DNA damage is the major factor contributing to CDDP cytotoxicity, many DNA repair systems, such as nucleotide excision repair (NER) and mismatch repair, have been identified to be involved in CDDP resistance in cancer cells [10]. Furthermore, platinum–DNA adduct formation can lead to DNA double-strand breaks (DSBs), which is the most lethal type of DNA damage. This DNA damage can trigger DNA damage response (DDR), an intricate signaling network composed of various DNA repair systems in cancer cells. Of the DNA repair pathways, homologous recombination (HR) repair is a major repair system responsible for DNA DSBs [11]. After DSB formation, HR repair is initiated by the essential effectors BRCA1 and BRCA2. These effectors work together to facilitate DSB end resection and promote the recruitment of RAD51 to the damage site. The formation of RAD51-coated filaments on DNA can induce DNA strand repair by searching the homologous chromatid. Notably, tumor cells with BRCA1 or BRCA2 dysfunction are susceptible to poly(ADP-ribose) polymerase (PARP) inhibitors [12, 13]. The primary function of DNA repair enzymes of the PARP family is DDR detection and initiation induced by DNA single-strand breaks (SSBs). Because the HR pathway is the major repair system of DSBs in cancer cells, the accumulation of SSBs caused by PARP inhibition can lead to increased DSB formation and subsequent cell death. Moreover, laboratory studies have demonstrated that the activity of the HR system is correlated with CDDP cytotoxicity in cancer cells [14–16]. Collectively, these results indicate that the HR pathway can be a feasible target in cancer treatment with DNA damage agents.

O6-methylguanine-DNA methyltransferase (MGMT) is a DNA repair enzyme that plays a role in protecting cells from cytotoxic effects of alkylating agents, especially those generating O6-alkylguanines [17]. This enzyme can repair DNA through the transfer of alkylating adducts from the O6 position of guanine in DNA to its cysteine residue (Cys145), the active site of this protein. Notably, we previously identified the prognostic value of MGMT expression levels in patients with NPC receiving CDDP-based CRT. In this study, high MGMT expression levels in NPC cells were correlated with low survival rates in patients treated with CDDP-based CRT [18]. Accumulating evidence is indicating that high MGMT expression levels can predict worse survival in patients with various types of cancers treated with platinum-based chemotherapy regimens [19–21]. In in vitro functional studies, we demonstrated that MGMT mediated CDDP-induced cytotoxicity and DNA repair activity in NPC cells [18]. Moreover, a study demonstrated that MGMT physically interacts with the molecular complex containing BRCA2 and is associated with BRCA2 degradation [22]. In mRNA expression analyses of profiled DNA damage-associated genes, we found that MGMT inhibition significantly reduced *RAD51* expression in NPC cells. These results suggest that MGMT may play a role in CDDP-induced DDR through involvement in HR signaling in cancer cells. Therefore, here, we investigated the molecular crosslinking between MGMT and the HR pathway and its clinical implications in NPC cells.

## Methods

### Cell culture

Human NPC cell lines, HONE-1 and TW01, were initially derived from patients with NPC [23, 24]. Topgen Biotechnology (Kaohsiung, Taiwan) authenticated these cell lines by using the short tandem repeat profile. These NPC cells were routinely culture as described previously [18].

### Antibodies and reagents

Monoclonal anti-MGMT antibodies were obtained from LTK BioLaboratories (Taoyuan, Taiwan). Chemical agents including O6-benzylguanine (O6BG) and olaparib and antibodies targeting BRCA1, pBRCA1 (Ser988), BRCA2, RAD51, β-actin, and lamin A/C were purchased from Santa Cruz Biotechnology (Dallas, TX, USA). Monoclonal anti-γ-H2AX antibodies were purchased from Cell Signaling Technology (Danvers, MA, USA). Other experimental reagents used are listed in a previous report [18].

### Human DNA damage signaling gene profiling

The relative mRNA expression of genes involved in DNA damage signaling were examined using an RT^2^ Profiler PCR array (Catalog No. PAHS-029Z, Human DNA Damage Signaling Pathway, Qiagen) according to manufacturer’s protocol. In brief, after HONE- cells were treated with or without O6BG (120 μM) for 8 h, total RNA was extracted using Qiagen columns (Qiagen, Valencia, CA, USA) and reverse transcribed using the SuperScript First Strand Synthesis System (Invitrogen Life Technologies). After the cDNA was applied to the Profiler PCR array, real-time PCR was performed using the ABI 7500 sequence detection system (Applied Biosystems) and PCR master mix (SA Biosciences RT^2^ qPCR Master Mix; Qiagen) for SYBR Green detection. Samples were amplified under the following conditions: a precycling hold at 95 °C for 5 min, 40 cycles of denaturation at 95 °C for 15 s and annealing at 60 °C for 1 min. Changes in mRNA expression were analyzed using ΔΔCt method and quantified by expression normalization with some housekeeping genes (*ACTB*,* B2M*, and* GAPDH*).

### Western blot analysis

Western blot analyses were performed as described previously [18]. In brief, cells were lysed in lysis buffer (Merck Millipore, Darmstadt, Germany) and then the extracted proteins were separated through sodium dodecyl sulfate polyacrylamide gel electrophoresis and subsequently transferred onto polyvinylidene fluoride membranes (Merck Millipore, Darmstadt, Germany). The nuclear proteins were extracted using a commercial fractionation kit (Abcam, Cambridge, UK) according to the manufacturer’s protocol. The immunoreactive signals of the membranes were detected using the Western Lightning Plus-ECL Enhanced Chemiluminescence Substrate (PerkinElmer, Inc., Waltham, MA, USA) and KodakX-Omat film (Kodak, Chalon/Paris, France). The band densities of immunoblotting were quantified using ImageJ densitometry analysis and normalized to β-actin or lamin A/C protein levels.

### Quantitative real-time PCR

For quantitative real-time PCR (qPCR), NPC cells were lysed in TRIzol reagent (Invitrogen, Carlsbad, CA, USA). Total RNA was isolated using Qiagen columns (Qiagen, Valencia, CA, USA) and reverse transcribed using the SuperScript First Strand Synthesis System (Invitrogen Life Technologies). TaqMan gene expression assay kits for RAD51 (Hs00947967_m1) were purchased from Applied Biosystems (Carlsbad, CA, USA). The relative mRNA levels of *RAD51* were detected using the ABI 7500 sequence detection system (Applied Biosystems) and calculated using the ΔΔCt method, with *GAPDH* mRNA as an endogenous control.

### Transient knockdown using small interfering RNA transfection

For MGMT silencing, small interfering RNA (siRNA) duplexes were designed to target two separate coding regions: 5ʹ-AAGCTGGAGCTGTCTGGTTGT-3′ (nucleotides 52–71) and 5′-AAGGTTGTGAAATTCGGAGAA-3′ (nucleotides 310–330). For nontarget silencing, the siRNA sequence targeting the coding region 5′-GCCATTCTATCCTCTAGAGGATG-3′ of luciferase was designated. NPC cells in the exponential growth phase were transfected with the siRNA duplex using Lipofectamine 2000 (Thermo Fisher Scientific, Waltham, MA, USA) according to the manufacturer’s instructions. Detailed siRNA transfection conditions were described elsewhere [18].

### Correlation analyses using data from the Gene Expression Omnibus database

For correlation analysis of gene expression levels, the clinical transcriptomes of NPC tumors were obtained from the Gene Expression Omnibus database (accession GSE102349) by using the Illumina HiSeq 2000 platform. This NPC cohort comprised 113 fresh tumor specimens with no treatment [25]. We analyzed the correlation between *MGMT* and *RAD51* expression levels by using Pearson correlation analyses.

### Immunoprecipitation assay

Co-immunoprecipitation (Co-IP) analyses were conducted according to a previous report [26]. To exclude the contaminating effect of DNA attached to tested proteins, 20 U/ml of DNase I (Roche) was added in lysis buffer. In brief, the cell lysates were subsequently sonicated, washed, and incubated with anti-MGMT antibodies (Abcam) or negative control IgGs (Santa Cruz Biotechnology). After incubation for 24 h, Pierce Protein A/G UltraLink Resin (Thermo Fisher Scientific) was added to capture immune complexes. After washing, the precipitated proteins were resuspended in nonreducing loading buffer and heated at 95 °C for 5 min before Western blot analyses.

### Immunofluorescence assay

The immunofluorescence analyses were performed according to two previous reports [26, 27]. After the indicated treatment, NPC cells were fixed in 4% paraformaldehyde for 20 min, permeabilized with a 0.05% Triton X-100 in TBS for 20 min, and then blocked with 3% BSA for 1 h. The NPC cells were then incubated with specific antibodies at 4 °C overnight. The dilution conditions for the antibodies used were as follows: 1:200 for anti-MGMT, 1:500 for anti-BRCA1 and anti-γH2AX, and 1:1000 for anti-RAD51 antibodies. Specific secondary antibodies, Alexa-488 and Alexa-594 (Thermo Fisher Scientific, Waltham, MA, USA), were diluted at 1:1000 and incubated at room temperature for 1 h. Nuclei were stained with DAPI (Sigma-Aldrich, St. Louis, MO, USA) at room temperature for 10 min. Cells were mounted with Dako fluorescent mounting medium (Agilent Technologies, CA, USA), and the images were captured on an Olympus FV1000 Laser Confocal Microscope. The fluorescence intensities within NPC cells were analyzed using the FV10-ASW 4.0 viewer.

### Proximity ligation assay (PLA)

After CDDP treatment (10 μM) for 24 h, HONE-1 cells were subjected to PLA as described previously [28]. In brief, tested cells were first fixed with paraformaldehyde for 15 min, and subsequently permeabilized with PBS containing Triton X-100 (Calbiochem) for 30 min. After permeabilization, tested cells were blocked in Blocking Solution (Sigma-Aldrich) at 37 °C for 1 h, and then incubated with primary anti-MGMT (LTK BioLaboratories, Taoyuan, Taiwan) and BRCA1 antibodies (Santa Cruz Biotechnology, Dallas, TX, USA) at 4 °C overnight. Following incubation with primary antibodies, tested cells were successively incubated with PLA probes in antibody diluent for at 37 °C for 1 h, ligation solution at 37 °C for 30 min, and amplification solution at 37 °C for 100 min. After the addition of Duolink in situ mounting medium (Sigma-Aldrich) and DAPI for 10 min, immunofluorescence images were captured with a confocal microscope (FV1000, Olympus).

### Neutral comet assay

The neutral comet assay was performed as described previously [28]. In brief, after being subjected to the indicated treatment, NPC cells were mixed with 100 μL of 1.5% low-melting point agarose and pipetted onto microscopic slides coated with 100 μL of 1% normal melting point agarose at 4 °C for 15 min. After gel adhesion, the cell slides were treated with neutral lysis solution for 20 min. Electrophoresis was performed using the neutral electrophoresis buffer at 25 V (current 300 mA) for 20 min. After electrophoresis, the cell slides were incubated in a neutralizing buffer for 20 min. The cell slides were then stained with propidium iodide (PI) and examined under a fluorescence microscope (Nikon, Optiphot-2, Tokyo, Japan) at 40 × magnification. Microscopic images of the cell tails were scored using CometScore (TriTek, Sumeduck, VA, USA).

### Measurement of HR activity

HR activity was analyzed using a PCR-based HR assay kit (Norgen Biotek, ON, Canada) according to the manufacturer’s instruction. In brief, NPC cells were first transfected with two plasmids containing different mutations in their lacZ coding region for 24 h and then treated with O6BG at indicated concentrations for 8 h. For siRNA-transfected NPC cells, HR reporting plasmids were cotransfected for 24 h. For the PCR reaction, a set of universal primers amplifying all plasmid DNA was used as control for transfection efficiency. Another set of primers amplifying plasmid DNA generated by HR was used to measure repair activity. The level of recombinant DNA produced in experimental cells was analyzed using the ΔΔCt method and expressed as a ratio to the control cells.

### Xenograft studies

All animal experiments were approved by the Institutional Animal Care and Use Committee of Chi-Mei Medical Center (approval number: 102120606) and conducted in accordance with the Guide for the Care and Use of Laboratory Animals. At first, 2 × 10^6^ HONE-1 cells mixed with Matrigel (Collaborative Research) were subcutaneously inoculated in the right flanks of male BALB/c nude mice (BioLASCO Co., Ltd, Taipei, Taiwan). The tumor volume (V, mm^3^) was calculated as V = π/6 × length (mm) × width^2^ (mm^2^). When the size of growing tumors reached ≥ 100 mm^3^, the indicated treatment was started in the NPC xenografts. The study xenografts were randomized into four groups (n = 6 in each group): (a) daily intraperitoneal (i.p.) injections of 1 × PBS (control group), (b) twice-weekly i.p. injections of CDDP (3 mg/kg), (c) daily i.p. injections of O6BG (2.5 mg/kg), and (d) i.p. injections of a combination of CDDP (two times/week) and O6BG (daily). Treatments were continued until the subcutaneous tumor size became approximately 2000 mm^3^.

### Immunohistochemical studies of xenografts

After the mice were sacrificed, primary tumors in the flank were excised and weighed. Immunohistochemistry (IHC) staining procedures were performed based on a previous report [18]. Tumor tissues were routinely fixed in formalin and embedded in paraffin. The paraffin-embedded tissue blocks were then cut into 3-mm-thick tissue slices. After routine deparaffinization and rehydration, slides were heated using a microwave treatment in citrate buffer (pH 6) for 7 min for antigen retrieval. Endogenous peroxidase was quenched using a 3% H_2_O_2_ treatment. After a wash, slides were incubated with anti-MGMT, anti-RAD51, and anti-pBRCA1 antibodies for 1 h, followed by secondary antibody incubation and hematoxylin counterstaining.

### Cell viability and clonogenic assay

The cell viability and clonogenic assay were performed using methylene blue staining as previously described [26]. For the viability assay, NPC cells were initially seeded at 1 × 10^4^ to 2 × 10^4^ cells/well into 24-well plates overnight for adhesion, and then subjected to the indicated treatment. For the clonogenic assay, NPC cells were first seeded at 5 × 10^3^ to 8 × 10^3^ cells/well into 6-well plates overnight, followed by the indicated treatment for 24 h. After a cell wash, the tested cells were incubated in treatment-free medium until colonies developed.

### Cell apoptosis analysis through annexin V and PI staining

A commercial annexin V-FITC apoptosis detection kit (Thermo Fisher Scientific) was used to evaluate cell apoptosis as described previously [18]. NPC cells were first seeded in 10-cm^2^ cell culture plates at a density of 1 × 10^6^ to 2 × 10^6^ cells per plate, followed by the indicated treatment when the cell densities reached 70–80% confluency. Following treatment, cells were routinely trypsinized, washed, and resuspended in annexin V staining buffer according to the manufacturer’s protocol. After incubation for 20 min at room temperature in the dark, the annexin V and PI staining intensities of the test samples were detected using a FACS Calibur flow cytometer (BD Biosciences) and analyzed using CellQuest (BD Biosciences).

### Statistical analysis

All assays were conducted at least in triplicate. The results, presented as means ± their standard deviations (SDs), were compared using Wilcoxon rank-sum test on SPSS (version 20.0; IBM Corp, Armonk, NY, USA). Statistically significant differences were considered at *P* < 0.05.

## Results

### MGMT correlates with RAD51 expression in NPC cells

**Fig. 1:**
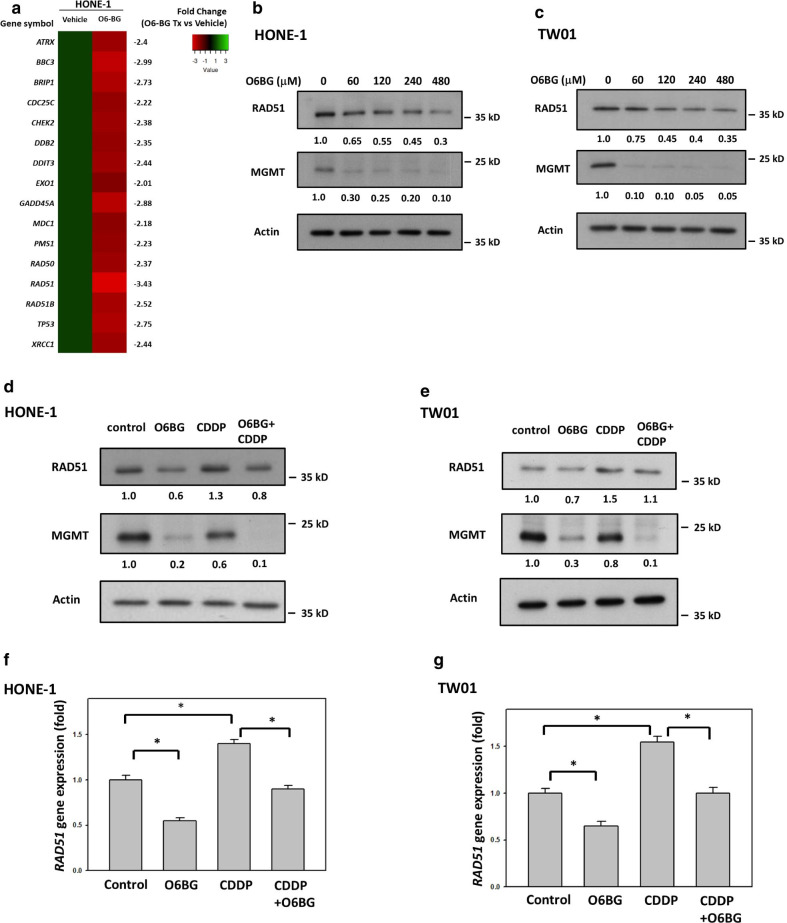
MGMT inhibition suppressed CDDP-induced RAD51 expression in NPC cells. **a** A heat map showing the expression changes of DNA repair genes with statistical significance, by using RT^2^ Profiler PCR Array, in HONE-1 cells with or without O6BG treatment, respectively. After HONE-1 cells were treated with O6BG (120 μM) for 8 h, the extracted mRNA was subjected to gene expression analyses. **b** HONE-1 and **c** TW01 cells were treated with O6BG at indicated concentrations for 8 h. **d** HONE-1 and **e** TW01 cells were treated with O6BG (60 μM), CDDP (10 µM), or a combination treatment for 8 h. The IC_50_ concentration of O6BG in both HONE-1 and TW01 cells was 120 µM. After the indicated treatment, cell lysates were analyzed using Western blotting. Fold changes in protein levels listed under each blot were normalized to the levels of the actin control. Following single-drug or combination treatment as indicated in **d** and **e**, the mRNA levels of **f** HONE-1 and **g** TW01cells were quantified using qPCR. Representative results of at least three independent experiments are presented. Bar values are presented as mean ± SD of at least three independent experiments. *P < 0.05

To evaluate the molecular interaction between MGMT and other DNA repair effectors, we first examined the expression levels of 84 genes involved in the DNA repair pathway using RT^2^ Profiler PCR Arrays in HONE-1 cells. If the cut-off point was defined as the gene expression fold change of more than ± 2 and *P* value < 0.0001, we identified 16 DNA repair-associated genes differentially expressed in NPC cells with pharmacological MGMT inhibition (O6BG treatment). These 16 identified genes were all downregulated by O6BG treatment, and fold-change of *RAD51* expression was the largest among expression changes of these genes (Fig. 1a). To further determine the correlation between MGMT expression and the HR signaling pathway, we investigated the expression levels of HR-associated proteins, including BRCA1, BRCA2, and RAD51, in NPC cells with O6BG treatment. In addition to BRCA2 (Additional file 1: Fig. S1), O6BG reduced RAD51 expression levels in NPC cells in a concentration-dependent manner. After treatment with 4 × IC_50_ concentrations of O6BG (480 μM) for 8 h, RAD51 expression levels were 30% and 35% in HONE-1 and TW01 cells, respectively, compared with control cells (Fig. 1b, c). To elucidate the interlinking of MGMT and HR activity in CDDP-induced DNA damage repair, we investigated the correlation between MGMT and RAD51 expression levels in NPC cells treated with CDDP. As shown in Fig. 1d, e, Western blot analyses revealed that CDDP treatment (10 µM) increased RAD51 expression levels in HONE-1 and TW01 cells, compared with control cells. However, cotreatment with O6BG (at 1/2-fold of IC_50_ concentration) reduced CDDP-induced RAD51 expression in these two NPC cell lines. To determine the regulatory mechanisms underlying the effects of MGMT inhibition on RAD51 expression, we examined *RAD51* mRNA levels in NPC cells. RT-PCR analyses revealed that CDDP treatment increased *RAD51* mRNA levels to 140% and 155% in HONE-1 and TW01 cells, respectively, whereas O6BG treatment reduced *RAD51* mRNA levels to 55% and 60% in HONE-1 and TW01 cells, respectively, compared with control cells (Fig. 1f, g). Moreover, in combination with CDDP treatment, O6BG treatment reduced *RAD51* mRNA levels from 140% to 90% and 155% to 100% in HONE-1 and TW01 cells, respectively, compared with control cells. These results suggest that MGMT inhibition can suppress *RAD51* expression levels in NPC cells with CDDP treatment.

**Fig. 2:**
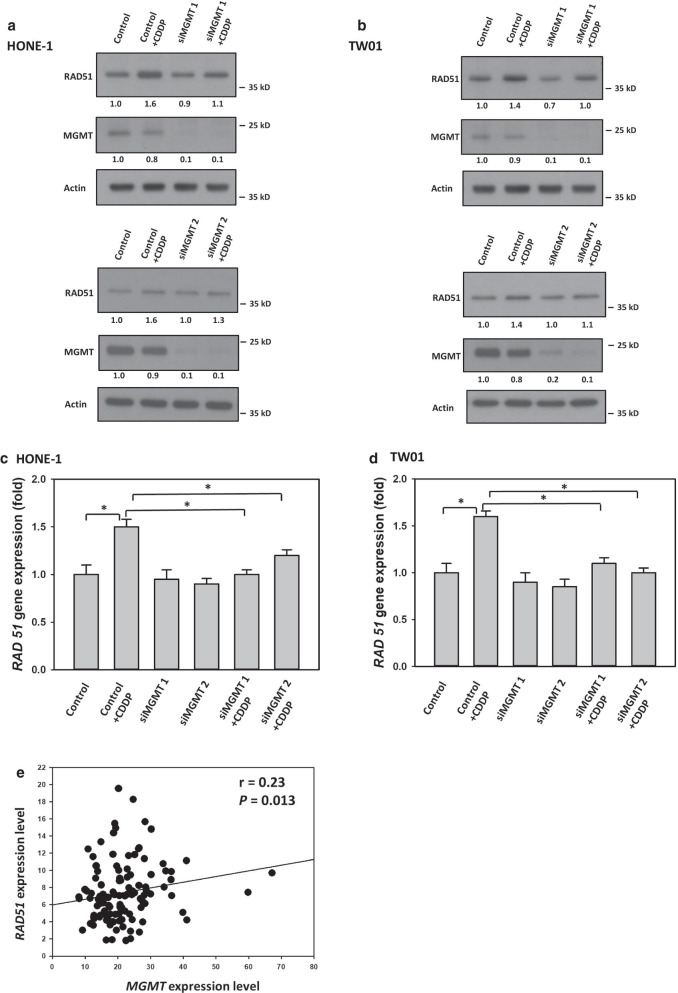
MGMT modulated CDDP-induced RAD51 expression in NPC cells. **a** HONE-1 and **b** TW01 cells transfected with scrambled or two independent *MGMT*-targeted siRNAs were treated with CDDP for 8 h. Fold changes in protein levels listed under each blot were normalized to the levels of the actin control. Following treatment with CDDP as indicated in **a**, **b**, the mRNA levels of **c** HONE-1 and **d** TW01cells transfected with scrambled or *MGMT*-targeted siRNA were quantified using qPCR. Representative results of at least three independent experiments are presented. Bar values are presented as mean ± SD of at least three independent experiments. *P < 0.05. **e** The correlation between *MGMT* and *RAD51* expression levels in patients with NPC (GSE102349) was analyzed using Pearson correlation analyses. The correlation coefficient and *P* are indicated

To evaluate the interaction between MGMT and RAD51 further, we examined whether MGMT itself plays an active role in RAD51 expression regulation in NPC cells, especially with CDDP treatment. For this purpose, we used the siRNA technique to silence *MGMT* expression in NPC cells. As shown in Fig. 2a, b, Western blot analyses of our NPC cells revealed that specific-targeting siRNA transfections effectively reduced MGMT expression levels. Additionally, Western blot analyses demonstrated that RAD51 expression levels were lower in *MGMT*-deficient NPC cells treated with CDDP compared with *MGMT*-proficient cells (Fig. 2a, b). RT-PCR analyses also showed that *RAD51* mRNA levels were decreased by 40%–50% and 50%–55%, respectively, in *MGMT*-deficient HONE-1 and TW01 cells treated with CDDP, compared with *MGMT*-proficient cells (Fig. 2c, d). To evaluate the correlation between *MGMT* and *RAD51* expression in patients with NPC, we reappraised the data deposited in one public dataset (GSE102349). An examination of gene expression levels in a total of 113 patients with NPC revealed that *MGMT* expression was positively correlated with *RAD51* expression, as shown in Fig. 2e. Taken together, these results suggest that MGMT correlates with RAD51 expression levels in NPC cells, particularly with CDDP treatment.

### MGMT interacts with BRCA1 in NPC cells treated with CDDP

**Fig. 3:**
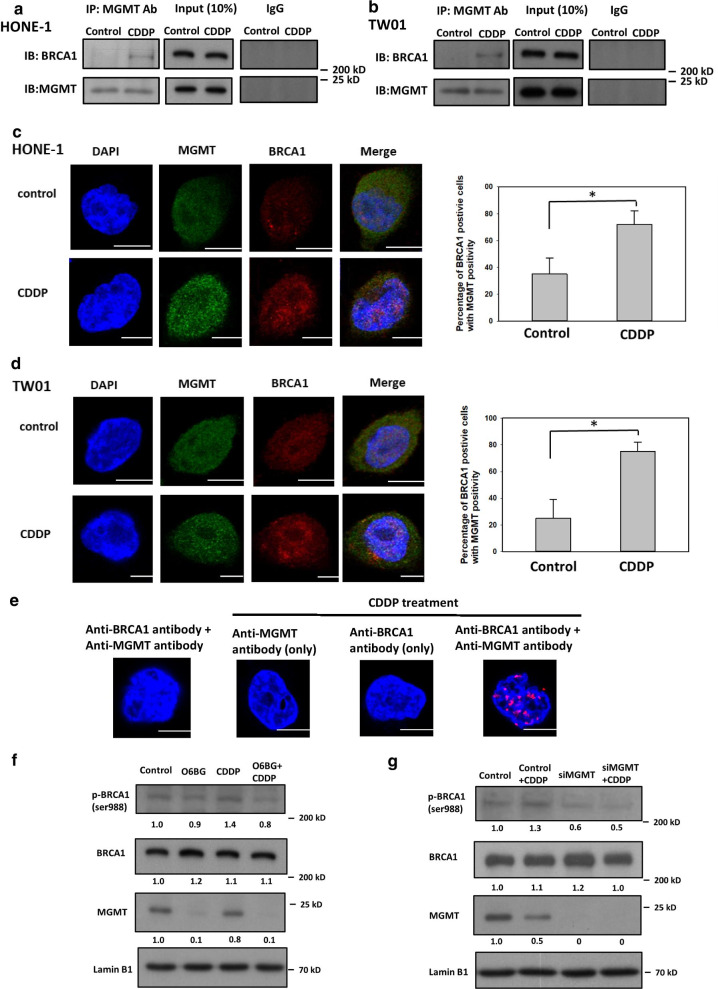
MGMT interacted with BRCA1 in NPC cells with CDDP treatment. After treatment with or without 10 µM CDDP for 8 h, the protein lysates of **a** HONE-1 and **b** TW01 cells were subjected to Co-IP analyses with 1 µg/mL anti-MGMT antibodies followed by Western blotting. Following the indicated treatment as in **a** and **b**, representative immunofluorescence images of DAPI, MGMT, and BRCA1 in **c** HONE-1 and **d** TW01 cells were processed through confocal microscopy. At least 200 cells were subjected to each treatment in each experiment. The bar graph (right panel) shows the percentage of tested cells containing five or more colocalizing foci for the staining proteins. Bar values are presented as mean ± SD of at least three independent experiments. *P < 0.05. **e** Endogenous physical interactions between MGMT and BRCA1proteins were detected by using in situ PLA in HONE-1 cells with or without CDDP treatment (10 µM for 8 h). PLA-positive signals are indicated by red fluorescent puncta and visualized by fluorescence microscopy. DAPI was used to detect the nuclei. To examine nuclear proteins, HONE-1 cells were initially **f** treated with O6BG (60 μM) or **g** transfected with *MGMT*-targeted siRNA. Following CDDP treatment as indicated in **a**, nuclear protein lysates were extracted and subjected to Western blot analyses with anti-pRBCA1, BRCA1 and MGMT antibodies. Fold changes in protein levels listed under each blot were normalized to the levels of the actin control. Representative results of at least three independent experiments are shown. Scale bars indicate 10 µm

A previous report demonstrated that MGMT physically interacts with BRCA2 in human cells [22]; however, the interactions between MGMT and other HR-associated components remain unclear. Therefore, we first used Co-IP analyses to evaluate the interaction between MGMT and BRCA2 in NPC cells, which revealed an increase in the amount of BRCA2 conjugated with anti-MGMT antibodies in NPC cells treated with CDDP compared with control cells (Additional file 1: Fig. S2). Notably, after precipitation and blotting with anti-MGMT and BRCA1 antibodies, respectively, we also observed the presence of MGMT–BRCA1 conjugates in NPC cells (Fig. 3a, b). Furthermore, the amounts of MGMT–BRCA1 conjugates also increased in CDDP-treated NPC cells compared with control cells. To further determine whether MGMT can interact with BRCA1 in NPC cells, we used confocal microscopy for locus detection after immunofluorescence staining. After costaining with MGMT and BRCA1, we quantitated the number of NPC cells with double-staining positivity, which were defined by more than five loci of MGMT and BRCA1 coformation in the nuclei. The immunofluorescence studies revealed that CDDP treatment increased the percentages of HONE-1 and TW01 cells with double-staining positivity by 40% and 50%, respectively (Fig. 3c, d). For the definite detection of the physical interaction between MGMT and BRCA1, we performed PLA by using anti-MGMT and anti-BRCA1 antibodies as probes in HONE-1 cells. In this immunohistochemical tool that allows in situ detection of endogenous proteins, we found that PLA-positive signals were increased in NPC cells with CDDP treatment (Fig. 3e). These results all indicate that MGMT can physically interact with BRCA1 in NPC cells, especially with CDDP treatment.

Phosphorylation of BRCA1-specific residues is a critical step for DNA repair function of this protein. Among these specific residues, previous reports have demonstrated that phosphorylation of ser 988 on BRCA1 is involved in HR activation [29, 30]. To explore the biological significance of the crosstalk between MGMT and BRCA1, we examined the expression levels of this specific residue phosphorylation (ser 988) on BRCA1 in NPC cells. As shown in Fig. 3f, CDDP treatment increased the expression levels of ser 988 phosphorylation on BRCA1 in the nuclei of HONE-1 cells; by contrast, O6BG treatment reduced the expression levels of CDDP-induced ser 988 phosphoprotein in HONE-1 cells. After transfection with siRNA targeting MGMT, the expression levels of ser 988 phosphorylation on BRCA1 were also lower in *MGMT*-deficient cells treated with CDDP compared with *MGMT*-proficient cells (Fig. 3g). Accordingly, these findings suggest that MGMT suppression can disturb ser 988 phosphorylation on BRCA1 in NPC cells treated with CDDP.

### MGMT inhibition enhances CDDP-induced DNA damage and impairs HR activity in NPC cells

**Fig. 4:**
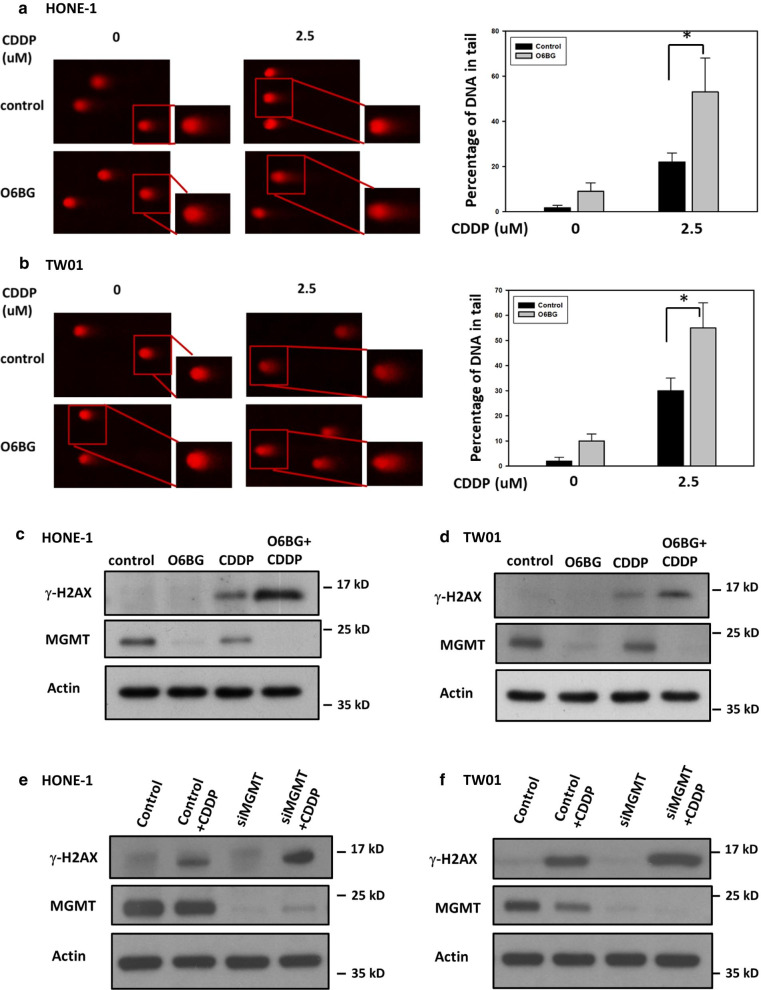
MGMT inhibition enhanced CDDP-induced DSB in NPC cells. After treatment with O6BG (60 μM), CDDP alone or a combination of both for 24 h, the DSB formation of **a** HONE-1 and **b** TW01 cells were analyzed using the neutral comet assay. After electrophoresis, the cell slides were stained with PI for visualization. The percentages of DNA in the tail were quantified by measuring the proportion of tail length in at least 100 comet cells and are presented as bar histograms. Bar values are presented as mean ± SD of at least three independent experiments. *P < 0.05. The expression of γ-H2AX was examined using Western blot analyses in **c** HONE-1and **d** TW01 cells following treatment with O6BG (60 μM), CDDP (10 µM), or a combination of both for 24 h. The expression of γ-H2AX induced by CDDP treatment was also examined in *MGMT*-proficient or -deficient NPC cells. After **e** HONE-1 and **f** TW01 cells were transfected with scrambled or *MGMT*-targeted siRNA for 24 h, these cells were treated with CDDP for another 24 h. Representative images of at least three independent experiments are shown

Because we found that MGMT is involved in RAD51 expression and BRCA1 phosphorylation in NPC cells treated with CDDP, we assessed whether MGMT expression can modulate CDDP-induced DSBs in NPC cells. To this end, we first performed a neutral comet assay to measure DSB formation in NPC cells. By measuring the tail movement of each cell, the comet assays demonstrated that O6BG treatment increased CDDP-induced DSB levels by 30% and 25% in HONE-1 and TW01 cells, respectively, compared with those with CDDP treatment alone (Fig. 4a, b). Moreover, combination treatment significantly increased the expression levels of γ-H2AX, a marker indicating DNA DSBs, compared with treatment with O6BG or CDDP alone (Fig. 4c, d). After pertinent siRNA transfection, the expression levels of γ-H2AX induced by CDDP treatment were increased in *MGMT*-deficient NPC cells compared with *MGMT*-proficient cells (Fig. 4e, f). All these findings suggest that MGMT can mediate CDDP-induced DSBs in NPC cells.

**Fig. 5:**
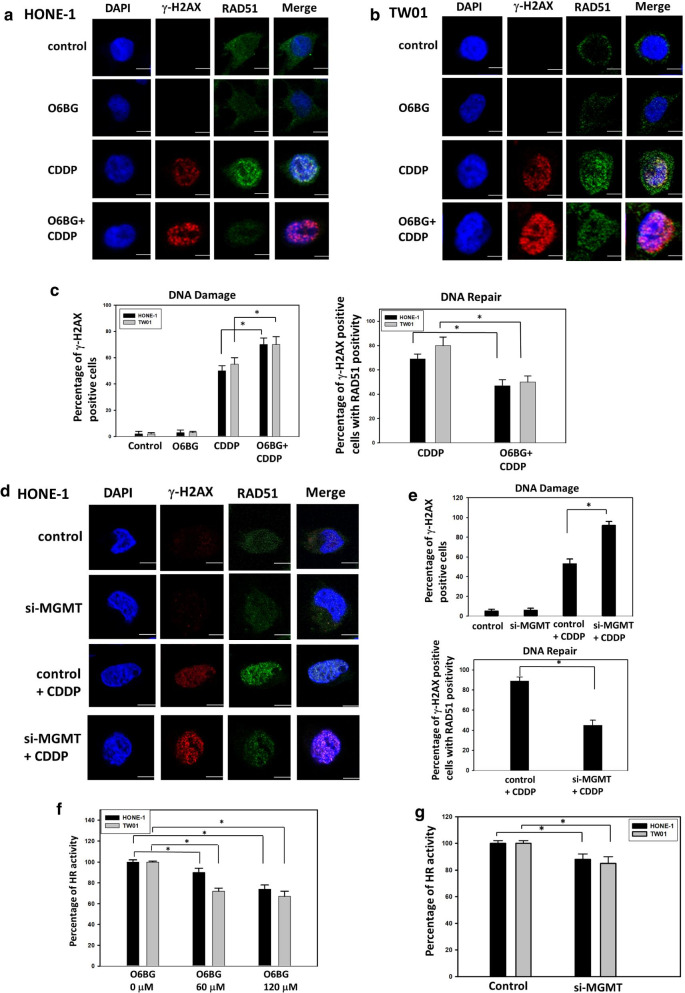
MGMT inhibition suppressed HR activity in NPC cells. The representative images of immunofluorescence foci of γ-H2AX and RAD51 in **a** HONE-1 and **b** TW01 cells are shown. Following treatment with O6BG (60 μM), CDDP (10 µM), or a combination of both for 24 h, the immunofluorescence foci of γ-H2AX and RAD51 were captured at ×60 on a confocal immunomicroscope. DAPI was used to detect the nuclei. Representative images of at least three independent experiments are shown. **c** The percentages of γ-H2AX-positive NPC cells in **a** and **b**, indicating the levels of DSB formation, were quantified in response to drug treatment (left panel). Only cells with more than five foci of γ-H2AX were considered positive. The percentages of γ-H2AX-positive cells with the recruitment of RAD51 foci in **a**, **b** were quantified in terms of DNA repair activity (right panel). Following CDDP or combination treatment, cells with more than five colocalized foci of γ-H2AX and RAD51 were considered positive. At least 100 cells per sample were counted in each experiment. **d** The representative images of immunofluorescence foci of γ-H2AX and RAD51 in *MGMT*-proficient or -deficient HONE-1 cells are shown. After cells were transfected with scrambled or *MGMT*-targeted siRNA for 24 h, these cells were treated with CDDP for another 24 h. The immunofluorescence foci of γ-H2AX and RAD51 were detected using confocal immunomicroscope as indicated in **a**. Scale bars indicate 10 µm. **e** The levels of DNA damage (upper panel) and DNA repair activity (low panel) in **d** were quantified by measuring the immunofluorescence foci of γ-H2AX and RAD51as indicated in **c**. Following treatment with the **f** indicated concentration of O6BG or **g** transfection of MGMT-targeted siRNA for 24 h, HR activities in the tested cells were determined using a plasmid-based recombination reporter assay. The value of the control cells was set as 100% after internalization with a backbone plasmid. Bar values are represented as mean ± SD of at least three independent experiments. *P < 0.05

To elucidate whether MGMT can modulate CDDP-induced DSBs in NPC cells by targeting HR repair activity, we analyzed the interaction between γ-H2AX and RAD51 through immunofluorescent staining, as described previously [27]. The results were comparable to those displayed in Fig. 4c, d; combination treatment with O6BG and CDDP increased the percentages of γ-H2AX-positive HONE-1 and TW01 cells, which contained more than five loci in the nucleus, by 25% and 20%, respectively, compared with CDDP treatment alone (Fig. 5a–c). Notably, through quantification of RAD51 recruitment in γ-H2AX-positive cells, combination treatment reduced HR repair activity by 25% and 30% in HONE-1 and TW01 cells, respectively, compared with CDDP treatment alone (Fig. 5c). In *MGMT*-deficient cells by using specific targeting siRNA, the percentages of γ-H2AX-positive cells were increased by 40%, whereas the percentages of cells with RAD51 recruitment in DNA damage site were decreased by 40% (Fig. 5d, e). These results suggest that *MGMT* suppression can attenuate CDDP-induced HR activity in NPC cells. We further assessed HR repair activity by using an in vivo plasmid recombination-based method. As shown in Fig. 5f, O6BG treatment reduced HR repair function in NPC cells in a dose-dependent manner. When treated with O6BG at a concentration of 120 μM, the HR activity levels were decreased by 26% and 33% in HONE-1 and TW01 cells, respectively, compared with control cells. Furthermore, HR activity was reduced by 10% and 15% in HONE-1 and TW01 cells after transfection with siRNA targeting *MGMT* compared with nontargeted control cells (Fig. 5g). Collectively, these results indicate that MGMT downregulation can enhance CDDP-induced DSBs and attenuate HR activity in NPC cells.

### Combination treatment with CDDP and MGMT inhibitor delays tumor growth in NPC xenografts

**Fig. 6:**
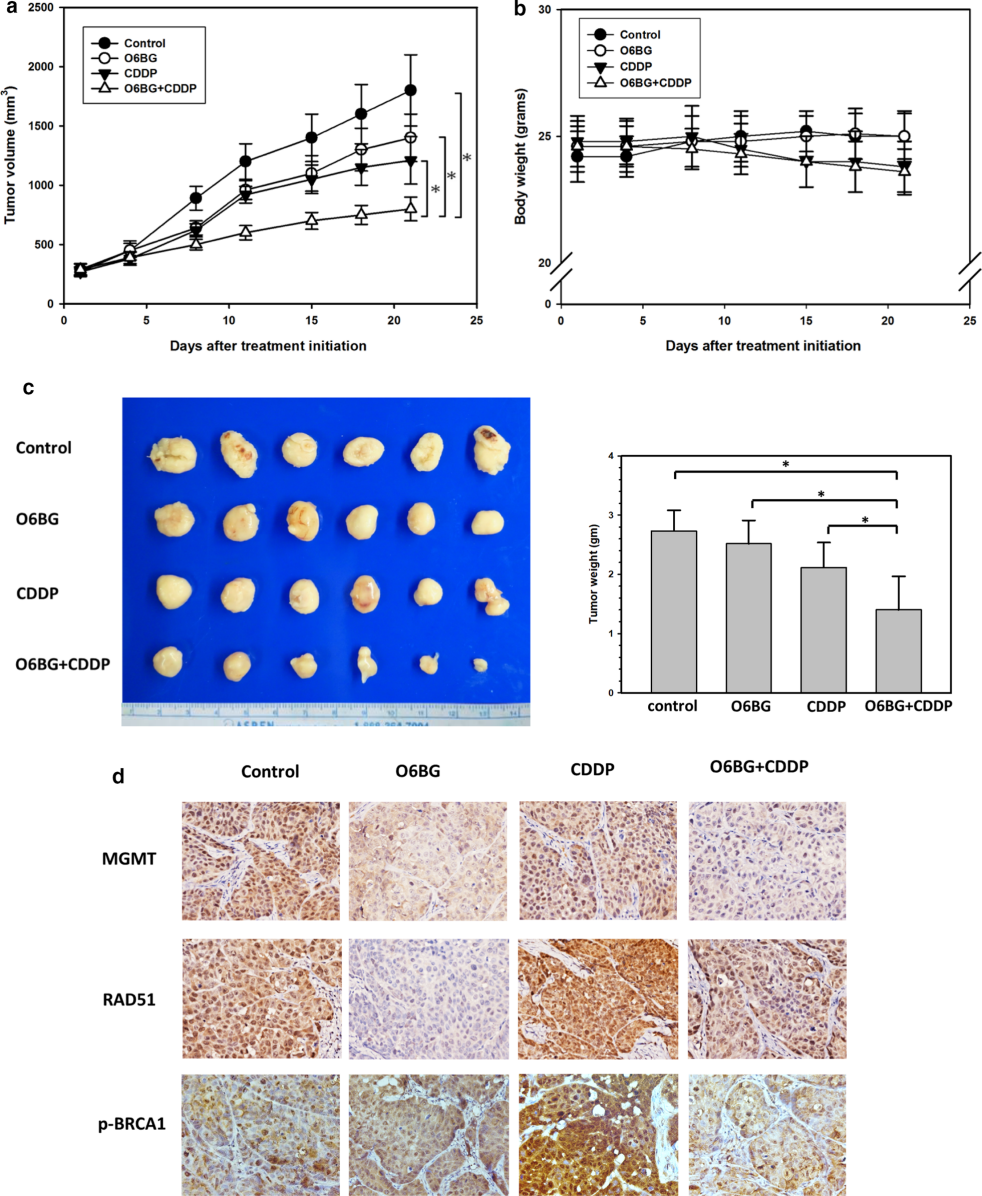
MGMT inhibition with CDDP treatment retarded NPC tumor growth. **a** Tumor volume and **b** body weight in mice bearing tumors were measured twice weekly. Tumor mass was measured with a caliper and calculated as π/6 × length (mm) × width (mm)^2^. **c** After 21 days of treatment, the mice were sacrificed, and tumor sizes were measured. The average tumor sizes in each treatment group are presented as a bar histogram (right panel). Bar values are presented as mean ± SD. *P < 0.05. **d** The expression of MGMT, RAD51, and p-BRCA1 was analyzed in paraffin-embedded tumor sections obtained from mice in each treatment group through immnunohistochemical staining. The slides were then examined under a fluorescence microscope, and photomicrographs were taken at a magnification of ×400

Because of the potential effectiveness of combination treatment with O6BG and CDDP reported in in vitro studies, we conducted tumor xenograft studies for further evaluation. We subcutaneously implanted HONE-1 cells in the flank of nude mice, and each treatment started when the tumor volume became palpable. Study xenografts were allocated to four treatment arms: vehicle control, daily O6BG injection, twice-weekly CDDP injection, and combination treatment with O6BG and CDDP. On treatment day 21, the tumor volume increased by 17.5-, 13.7-, 12-, and 7.5-fold in the vehicle, O6BG, CDDP, and combination treatment groups, respectively. The tumor volumes at the end of this xenograft study were significantly decreased in combination treatment group, compared with any other treatment (all *P* < 0.05; Fig. 6a). No significant changes in body weights were observed between mice receiving each treatment (Fig. 6b). The sizes of tumors dissected from sacrificed mice subjected to combination treatment were also significantly smaller than those of tumors in other treatment groups (Fig. 6c).

To assess alterations of HR components induced by O6BG in vitro, we examined the expression levels of RAD51and BRCA1 phosphorylation (ser 988) in tumors through IHC staining. Compared with control mice, RAD51 expression levels were significantly decreased in O6BG-treated tumors (Fig. 6d). Furthermore, in tumors subjected to combination treatment, the expression levels of RAD51 and BRCA1 phosphorylation were lower than those in tumors treated with CDDP alone. Taken together, these results support that MGMT inhibition can reduce CDDP-induced expression of RAD51 and BRCA1 phosphorylation in NPC cells. Further, combination treatment with MGMT inhibitor and CDDP can be efficacious against the growth of NPC cells.

### MGMT suppression enhances olaparib-induced cytotoxicity in NPC cells

**Fig. 7:**
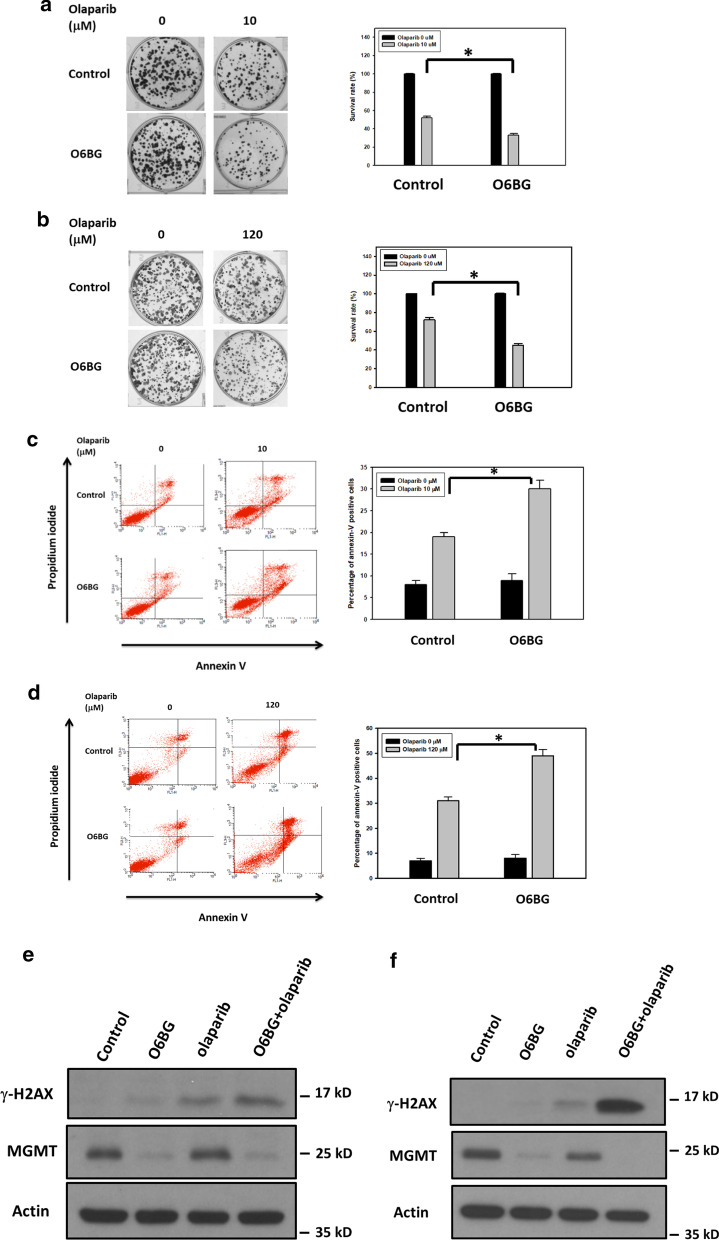
MGMT inhibition increased the cytotoxicity of PARP inhibitor in NPC cells. The survival rates of **a** HONE-1 and **b** TW01 cells were examined using the clonogenic assay. The percentages of apoptotic cells in **c** HONE-1 and **d** TW01 cell samples were analyzed through flow cytometry after costaining the cells with FITC-conjugated annexin V and PI. The γ-H2AX expression of **e** HONE-1 and **f** TW01 cells was investigated using Western blot analyses. Representative histograms indicated the percentages of colony formation and apoptosis cells (annexin V–positive cells). The tested cells were exposed to O6BG (60 μM), olaparib at indicated concentrations or combination treatment for 24 h, and then analyzed for colony formation, apoptotic cell proportion, and γ-H2AX expression. Experiments were performed at least three times. Bar values are presented as mean ± SD. *P < 0.05

Tumors harboring defects in the HR system is highly vulnerable to the cytotoxic effects of poly(ADP-ribose) polymerase (PARP) inhibitors [12, 13], and we investigated whether MGMT inhibition can increase the cytotoxicity of PARP inhibitors in NPC cells. After suppression of MGMT expression through the addition of O6BG for 8 h, the IC_50_ values of olaparib, the first PARP inhibitor in clinical use, were decreased by approximately 50% in both HONE-1 and TW01 cells (Table 1). The clonogenic assays also showed that the addition of O6BG enhanced the cytotoxicity of olaparib in NPC cells (Fig. 7a, b). After cotreatment with O6BG and olaparib, the numbers of forming colonies decreased by 22% and 27% in HONE-1 and TW01 cells, respectively, compared with treatment with olaparib alone. To further assess the cytotoxic effects of olaparib treatment in NPC cells with MGMT inhibition, we detected the percentages of apoptotic cells through FACS-based annexin V/PI double-staining. The flow cytometry analyses demonstrated that combination treatment with O6BG and olaparib increased the populations of apoptotic cells (annexin V–positive cells) by 13% and 19% in HONE-1 and TW01 cells, respectively, compared with treatment with olaparib alone (Fig. 7c, d). In addition, the expression levels of γ-H2AX were significantly increased in NPC cells treated with a combination of O6BG and olaparib compared with those treated with olaplarib alone (Fig. 7e, f). These results suggest that MGMT inhibition can enhance olaparib-induced cytotoxicity and DSBs in NPC cells.

**Table 1:** IC_50_ values of olaparib in NPC cells

	HONE-1 (µM)	TW01 (µM)
Control	10.5 ± 0.5	122.2 ± 8.2
O6BG	5.2 ± 0.4	61.5 ± 10.8

IC_50_ values of olaparib were determined using a cell viability assay. Cells in the exponential phase were first treated with O6BG (120 µM) for 8 h. After medium removal and cell washing, serial concentrations of olaparib were added for three generation times. Values are presented as means ± SDs of at least three independent experiments

To further examine whether MGMT expression levels mediated the anticancer effects of olaparib in NPC cells, the cytotoxicity tests of olaparib were conducted in TW01 cells with *MGMT*-targeted siRNA transfection. As shown in Additional file 1: Fig. S3a and b, the survival rates of *MGMT*-deficient TW01 cells treated with olaparib were decreased by 23%, whereas the populations of apoptotic cells were increased by 25%, compared with scrambled control. Western blot analyses also showed that olaparib treatment increased the expression levels of γ-H2AX in *MGMT*-deficient TW01 cells (Additional file 1: Fig. S3c). Collectively, our data indicate that MGMT expression levels modulate the cytotoxicity of PARP inhibitor in NPC cells.

## Discussion

Since the development of CDDP in the 1970s, this platinum-based chemotherapy drug has shown promising anticancer efficacy in a variety of cancers, including NPC. In addition to monotherapy, combination treatment with CDDP and other anticancer therapies, such as radiotherapy and other chemotherapy drugs, is also a common therapeutic strategy in cancer patients because of its synergistic effect against tumor growth [8, 9]. Even with the advances in immunotherapy, combination treatment with CDDP and an immune checkpoint inhibitor has demonstrated survival benefits in patients with a certain type of cancer, including head and neck cancer [8, 31, 32]. Accordingly, several researchers have devoted their research to overcoming CDDP resistance for decades. Currently, it is believed that CDDP resistance can be regulated by numerous intracellular (e.g., ion transporters, detoxification enzymes, and DNA repair pathway) and extracellular (e.g., hypoxia, acidity, and immunosuppressive cells) factors. Because the main cytotoxic effects of CDDP are generated from DNA damage in cancer cells, studies focusing on the action mechanisms of DDR in the regulation of CDDP resistance are compelling for most investigators. In fact, several reports have shown that platinum–DNA adducts can majorly be repaired by the activation of NER pathway [10]. If left unrepaired, the DNA damage caused by these bulky DNA adducts can progress to DSB formation, the repair of which is performed by the activation HR pathway [11]. Because genomic instability is a central part of tumor progression [33], the network of CDDP-induced DDR, which is composed of miscellaneous types of DNA pathways, is certainly intricate and entangled in cancer cells. Among various DNA repair pathways, MGMT is a unique DNA repair enzyme that repairs alkylating groups on the O6 position of guanine [17]. This DNA repair enzyme can repair O6-alkylguanines without the involvement of other repair proteins and undergo ubiquitination-mediated degradation after the repair process is completed. Although the action mechanisms remain unclear, several studies have reported that MGMT expression is correlated with CDDP cytotoxicity in cancer cells [34–36]. In our previous study, we have shown that MGMT can enhance the DNA repair activity for platinum–DNA adducts in NPC cells [18]. Furthermore, Philip and his colleagues have reported that MGMT is physically involved in BRCA2-containing molecular complexes in cancer cells [22]. Based on these results, we hypothesize that MGMT can modulate CDDP-induced DDR by targeting the HR pathway. In the present study involving several functional assays, xenograft studies, and data mining of a public database, we demonstrated that MGMT participated in the HR signaling pathway and affected CDDP and PRAP inhibitor cytotoxicity in NPC cells.

The HR signaling pathway has attracted considerable interest in cancer research because of the high cancer susceptibility arising from genomic mutations of its component proteins [30, 33]. This repair process involves initial damage recognition by the ataxia-telangiectasia mutated and ataxia telangiectasia and Rad3-related kinase; signal mediation by CHK2, BRCA1, and BRCA2; and final repair by the effector RAD51. Therefore, the function of RAD51 is critical to the HR repair efficiency and is correlated with CDDP resistance in cancer cells [37–40]. In the present study, we found that RAD51 expression levels were increased in NPC cells treated with CDDP, thus evidencing its important role in the repair of CDDP-induced DNA damage (Figs. 1, 2). Moreover, our study revealed that MGMT suppression by a specific inhibitor or the siRNA technique reduced CDDP-mediated RAD51 expression in NPC cells, comparable to the positive correlation data between *MGMT* and *RAD51* expression levels observed in a cohort of NPC patients (GSE102349). These results suggest that MGMT interacts with RAD51 expression in NPC cells, particularly after CDDP treatment. Because O6BG treatment inhibits MGMT expression through the ubiquitin–proteasome degradation pathway [17], some unknown biological effects may be involved in this process of MGMT inhibition. Therefore, prominent *RAD51* downregulation may be induced in NPC cells with O6BG treatment compared with those with siRNA transfection in the present study. In addition to RAD51, BRCA1 is essential to the signaling transduction of the HR pathway. Several reports have also demonstrated that patients with cancer frequently carrying *BRCA1* mutation are susceptible to CDDP treatment [41–44]. In addition to BRCA2, Co-IP, confocal immunofluorescence microscopy analyses and PLA studies revealed the physical interaction between MGMT and BRCA1 in NPC cells in the present study (Fig. 3). Interestingly, CDDP treatment significantly increased the amounts of immune complexes containing both MGMT and BRCA1 in NPC cells, also suggesting a role of MGMT in the CDDP-induced HR pathway. To further explore the biological significance of the interaction between MGMT and BRCA1, we examined whether MGMT can mediate BRCA1 phosphorylation (ser 988) in HONE-1 cells treated with CDDP. As noted, BRCA1 engages in the signaling transduction of the HR pathway mainly through the phosphorylation of its specific residues [29, 30]. The phosphorylation of ser 988 on BRCA1 mediated by CHK2 activation is required for the recruitment of RAD51 to the DNA damaged site. As expected, we found that CDDP treatment enhanced the expression levels of p-BRCA1 (ser 988) in the nucleus of HONE-1 cells, indicating its important role in the CDDP-mediated HR signaling pathway (Fig. 3). Additionally, we found that MGMT suppression (through O6BG treatment and the siRNA technique) abrogated CDDP-induced p-BRCA1 expression in HONE-1 cells. These results also indicate that MGMT is involved in BRCA1 signaling transduction of the HR pathway activated by CDDP treatment. In our previous study, we observed that MGMT proteins were present in nucleic acids containing platinum adducts [18]. Taken together, these results support the participation of MGMT in the signaling transduction of HR pathway induced by CDDP in NPC cells.

Because the main function of the HR pathway is DNA DSB repair, we subsequently investigated whether MGMT can determine CDDP-induced DSB formation in NPC cells. The neutral comet assay, an established technique for quantifying DSBs by measuring the tail length of a cell, was first used in our functional studies [41]. In this electrophoresis-based assay, cotreatment with O6BG and CDDP increased the tail lengths of NPC cells compared with CDDP treatment alone (Fig. 4). Comparably, CDDP-induced expression levels of γ-H2AX, a sensitive marker for DSB formation, were increased in O6BG-treated NPC cells. These results all suggest that MGMT is associated with CDDP-induced DSB regulation in NPC cells through the HR pathway. Therefore, we directly evaluated the efficiency of the HR pathway in repairing CDDP-induced DNA damage by using two established functional assays. One was an immunofluorescence-based study that considers the HR pathway to be activated if more than five colocalization foci of RAD51 and γ-H2AX immune signal are detected in the nucleus of the NPC cell [27]. The other was a reporter plasmid-based assay, which can measure HR activity by calculating the PCR product generated by this repair pathway [42]. In immunofluorescence-based studies, we found that cotreatment with O6BG and CDDP reduced the numbers of NPC cells with active HR signaling compared with CDDP treatment alone; in plasmid-based studies, MGMT suppression reduced the number of genomic products induced by the HR pathway (Fig. 5). Taken together, MGMT plays an active role in the HR signaling pathway responsible for the repair of CDDP-induced DNA damage in NPC cells.

MGMT is considered a perspective target in cancer therapy because of its important role in the protection of genomic damage from alkylating agents. Several therapeutic approaches have been pursued to inhibit MGMT expression in tumors, such as the utility of its pseudosubstrates, gene therapies, and viral proteins [17, 43]. The clinical efficacy of alkylating chemotherapy drugs in combination with specific MGMT inhibitor, such as O6BG, has been demonstrated in several trials [44–47]. In our xenograft study, combination treatment with O6BG and CDDP delayed NPC tumor growth compared with CDDP alone. Moreover, O6BG abolished CDDP-activated RAD51 and p-BRCA1 expression in tumor specimens (Fig. 6). These data support the participation of MGMT in the HR pathway and the therapeutic potential of O6BG when used in combination with CDDP. Although severe hematologic toxicities have been reported in clinical trials investigating the efficacy of combination treatment with O6BG and alkylating drugs, it is likely contributed by low MGMT expression levels in hematopoietic progenitor cells [17, 43]. However, the major toxicity caused by CDDP treatment is nephrotoxicity, and this treatment causes less hematological toxicity than treatment with other alkylating chemotherapy drugs [48]. Moreover, MGMT expression is typically higher in human liver, lung, and kidney tissues [17, 49]. Consequently, studies evaluating the clinical benefits of O6BG in combination with CDDP treatment in NPC patients are encouraged.

The HR pathway has become an emerging therapeutic target because of the successful development of PARP inhibition in cancer treatments [12, 13]. Based on the concept “synthetic lethality”, PARP inhibition can selectively lead to the death of BRCA1- or BRCA2-dysfunction cancer cells in which HR activity is deficient. The main function of PARP proteins is the recognition of SSB formation, and their specific inhibitors can trap these proteins on the DNA damaged site. When the replication machinery encounters these trapped proteins, the replication fork collapses with the formation of DNA DSBs. Accordingly, HR-deficient (HRD) tumor cells are highly sensitive to PARP inhibitors because of their incapability of efficiently repairing the excess formation of DNA DSB. Olaparib is the first PARP inhibitor in clinical use and is currently approved for use in the treatment of *BRCA*-mutated advanced ovarian, breast, pancreatic, and prostate cancers. Because MGMT inhibition attenuated HR activity, we tested the cytotoxic effects of a combination of O6BG and olaparib in the present study. Our functional assays revealed that O6BG increased olaparib-induced DSB formation and cell apoptosis in NPC cells, supporting the therapeutic strategy of combining MGMT and PARP inhibitor in treating cancer patients. Furthermore, in the modern era of precision medicine, identifying the most susceptible patients using predictive companion diagnostics is helpful to maximize the clinical benefit of a specific anticancer therapy. With the advances in the genomic sequencing technique, diagnostic approaches to recognizing HRD tumors have been developed for treatment response prediction of both CDDP and PARP inhibitors [50–52]. The regulation mechanisms of *MGMT* expression through its promoter methylation status have been well explored in patients with glioblastoma [17, 43]. In our previous study, we demonstrated that methylation modification of the *MGMT* promoter region by using high-throughput sequencing is also correlated with MGMT expression in NPC tumors [18]. Thus, the examination of the *MGMT* promoter methylation in gene signatures for identifying HRD tumors is notable.

## Conclusion

**Fig. 8:**
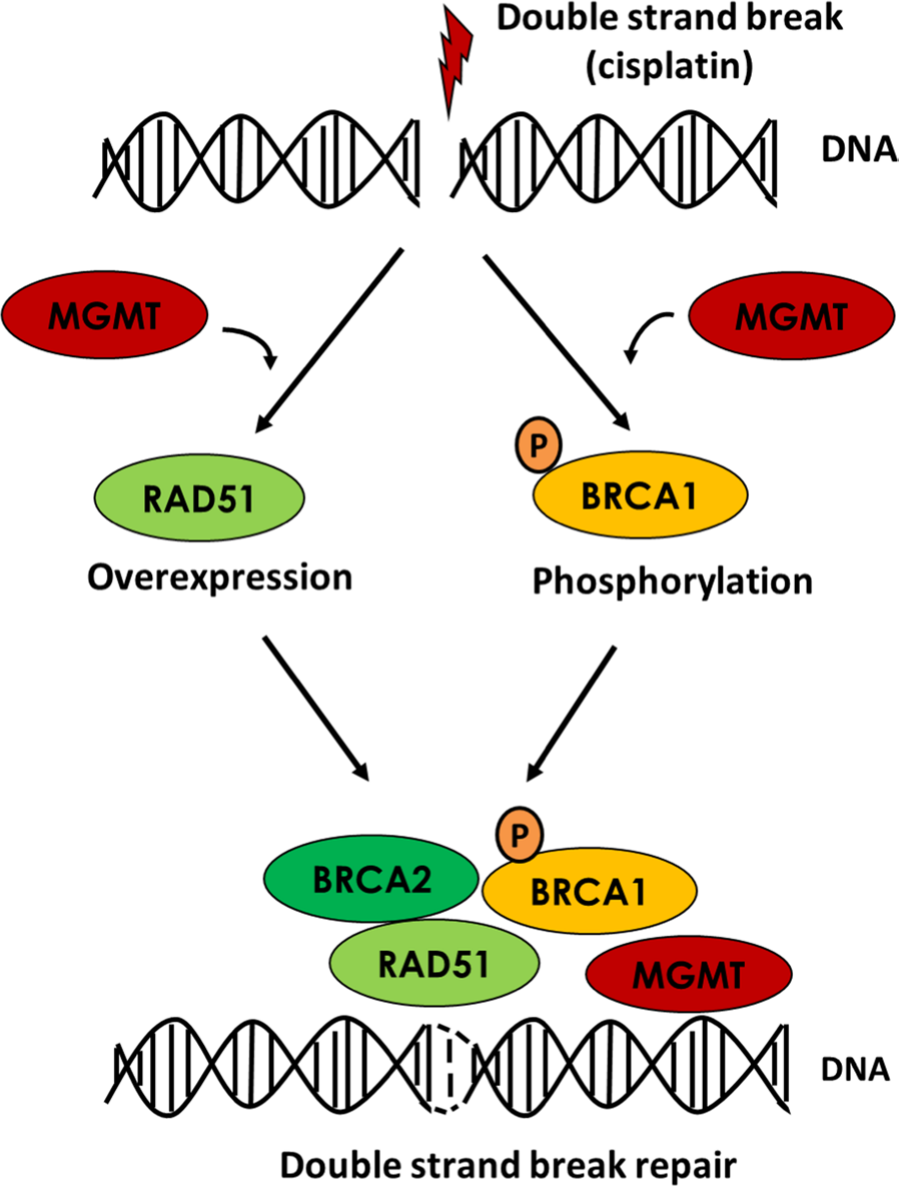
Schematic illustrating the role of MGMT in HR signaling in NPC cells. After the formation of platinum–DNA adducts, MGMT is involved in RAD51 and p-BRCA1 (ser 988) expression in HR signaling pathway. Through the participation in HR activation, MGMT could modulate CDDP and PARP inhibitor sensitivity in NPC cells

We demonstrated that MGMT is involved in RAD51 expression and BRCA1 phosphorylation in NPC cells (Fig. 8). By attenuating HR activity, the MGMT suppression enhanced DSB formation and cell death induced by CDDP or PARP inhibitor. These results suggest that MGMT is a potential therapeutic and diagnostic target in cancer treatment with CDDP or PARP inhibitor.

## Supplementary Information

**Additional file 1: Fig. S1.** BRCA1 and BRCA2 expression in NPC cells treated with O6BG. **a** HONE-1 and **b** TW01 cells were treated with indicated concentrations of O6BG for 8 h. The IC_50_ concentration of O6BG in both HONE-1 and TW01 cells was 120 μM. Cell lysates were subjected to Western blot analyses after the indicated treatment. Fold changes in protein levels listed under each blot were normalized to the levels of the actin control. Representative results of at least three independent experiments are shown. **Fig. S2.** MGMT interacted with BRCA2 in NPC cells treated with CDDP. After treatment with or without 10 μM CDDP for 8 h, the protein lysates of (A) HONE-1 and **b** TW01 cells were subjected to Co-IP analyses with 1 μg/mL of anti-MGMT antibodies, followed by Western blot analyses. Representative results of at least three independent experiments are shown. **Fig. S3.** MGMT mediated the cytotoxicity of PARP inhibitor in NPC cells. The **a** survival rates, **b** percentages of apoptotic cells, and **c** γ-H2AX expression of TW01 cells were examined using a clonogenic assay, annexin V staining, and Western blot analyses. TW01 cells transfected with scrambled or *MGMT*-targeted siRNA were treated with olaparib for 24 h. Representative histograms indicated the percentages of colony formation and apoptotic cells (annexin V–positive cells). Experiments were conducted at least three times. Bar values are presented as mean ± SD. *P < 0.05.

## Data Availability

The dataset generated or analyzed in this study is included with this article and can be made available from the corresponding author upon reasonable request.

## Abbreviations

NPC: Nasopharyngeal carcinoma
CDDP: Cisplatin
CRT: Chemoradiotherapy
NER: Nucleotide excision repair
DSB: Double-strand break
DDR: DNA damage response
HR: Homologous recombination
PARP: Poly(ADP-ribose) polymerase
SSB: Single-strand break
MGMT: O6-methylguanine-DNA methyltransferase
O6BG: O6-benzylguanine
q-PCR: Quantitative real-time PCR
siRNA: Small interfering RNA
Co-IP: Co-immunoprecipitation
PI: Propidium iodide
i.p.: Intraperitoneal
IHC: Immunohistochemistry
HRD: HR-deficient
