# Supplementary figures and images for: O6-methylguanine-DNA methyltransferase modulates cisplatin-induced DNA double-strand breaks by targeting the homologous recombination pathway in nasopharyngeal carcinoma
Source: J Biomed Sci. 2021 Jan 4;28:2. doi: 10.1186/s12929-020-00699-y (PMC7780675; doi:10.1186/s12929-020-00699-y)

## Slide 1
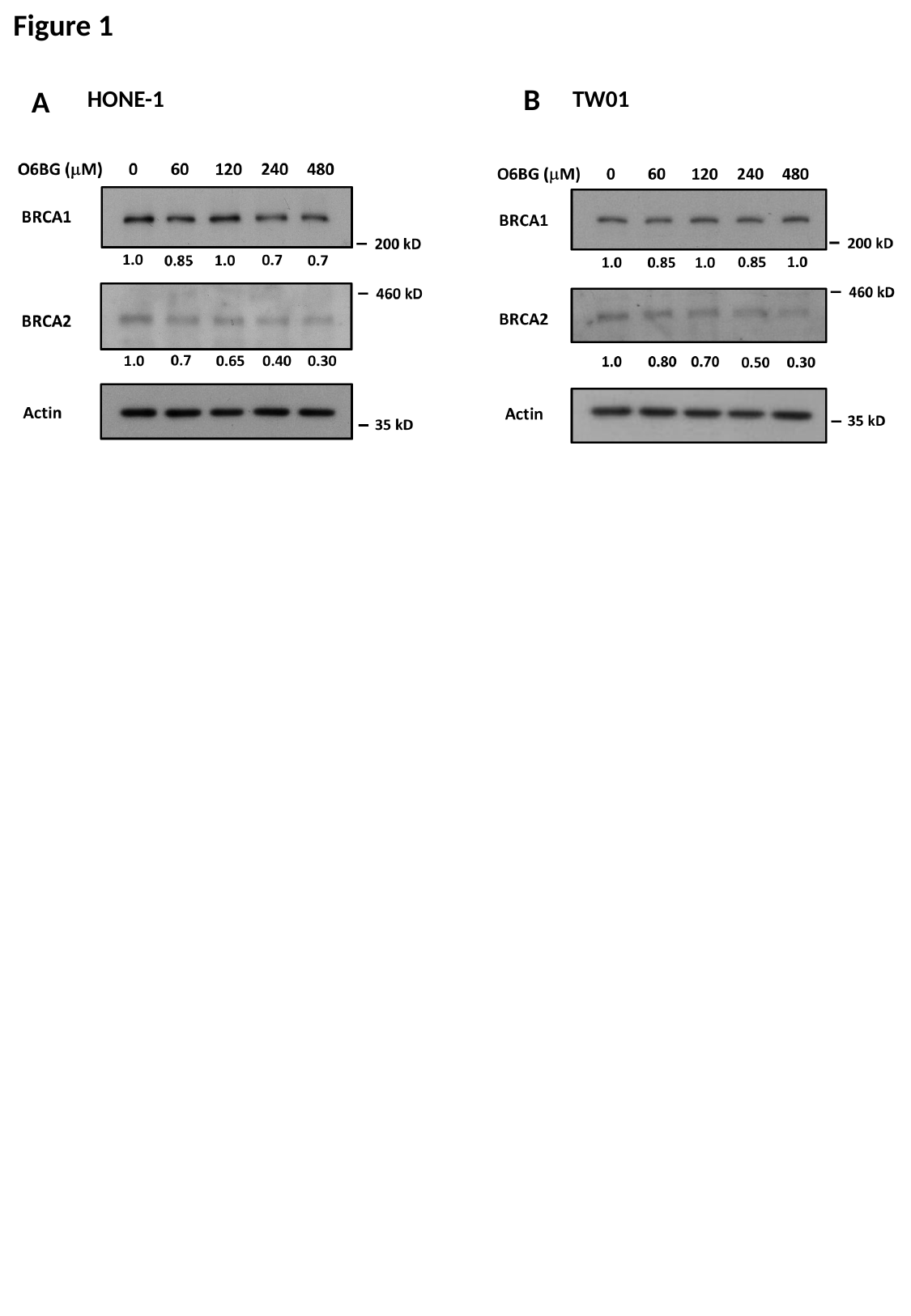

Figure 1
B
A
HONE-1
TW01

## Slide 2
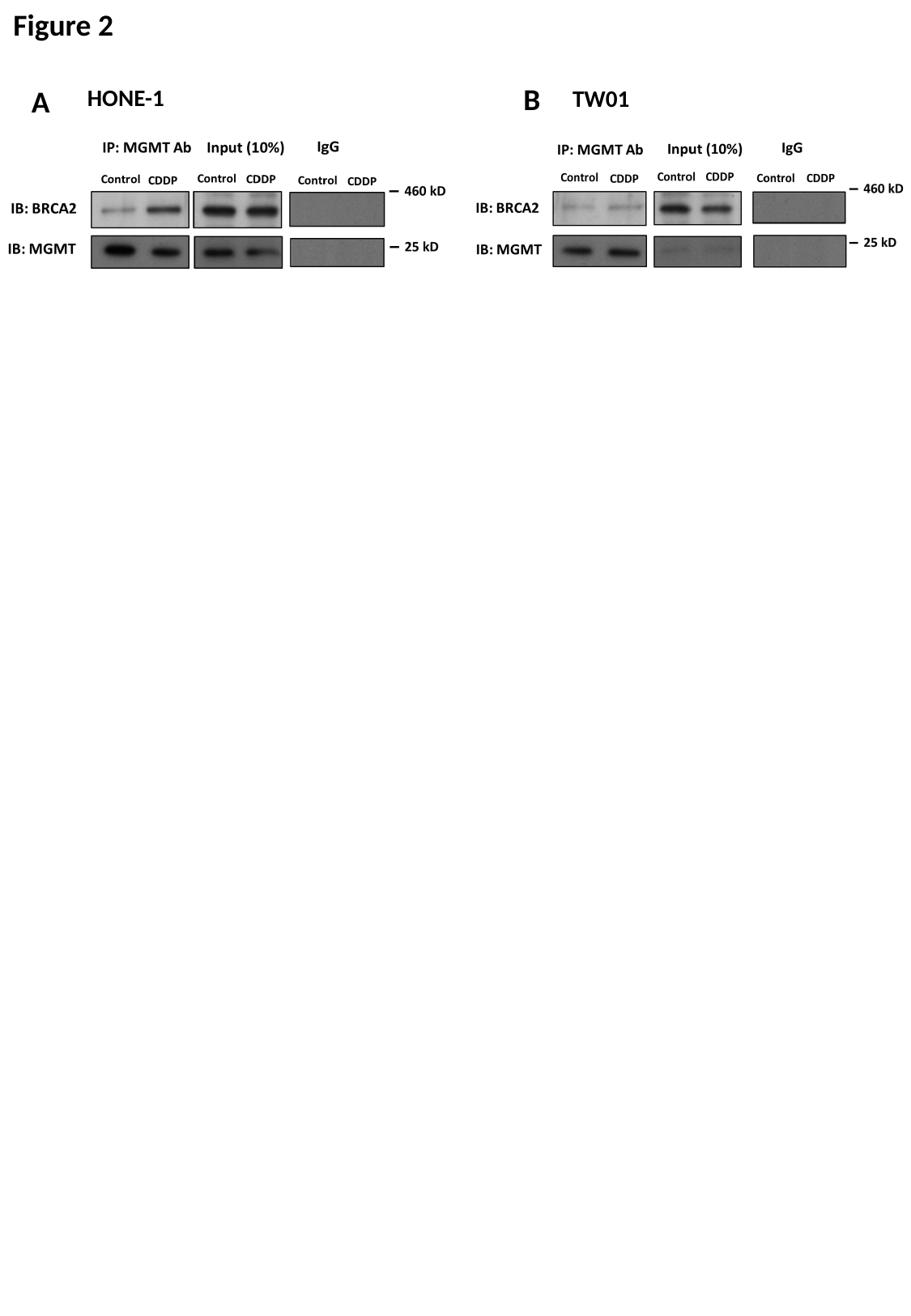

Figure 2
B
A
HONE-1
TW01

## Slide 3
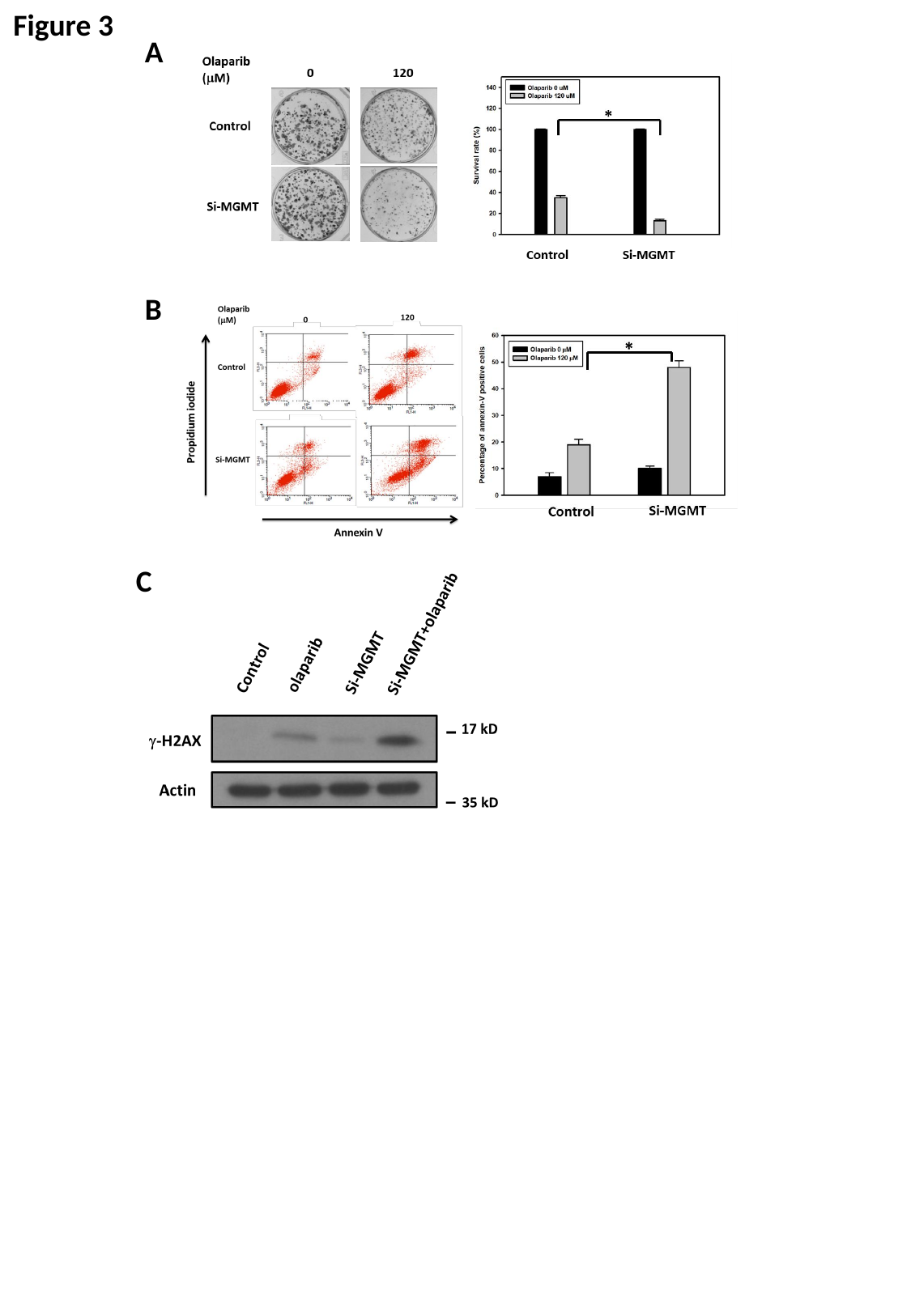

Figure 3
A
B
C

Supplement: Supplementary file 1 — Additional file 1: Fig. S1. BRCA1 and BRCA2 expression in NPC cells treated with O6BG. a HONE-1 and b TW01 cells were treated with indicated concentrations of O6BG for 8 h. The IC50 concentration of O6BG in both HONE-1 and TW01 cells was 120 μM. Cell lysates were subjected to Western blot analyses after the indicated treatment. Fold changes in protein levels listed under each blot were normalized to the levels of the actin control. Representative results of at least three independent experiments are shown. Fig. S2. MGMT interacted with BRCA2 in NPC cells treated with CDDP. After treatment with or without 10 μM CDDP for 8 h, the protein lysates of (A) HONE-1 and b TW01 cells were subjected to Co-IP analyses with 1 μg/mL of anti-MGMT antibodies, followed by Western blot analyses. Representative results of at least three independent experiments are shown. Fig. S3. MGMT mediated the cytotoxicity of PARP inhibitor in NPC cells. The a survival rates, b percentages of apoptotic cells, and c γ-H2AX expression of TW01 cells were examined using a clonogenic assay, annexin V staining, and Western blot analyses. TW01 cells transfected with scrambled or MGMT-targeted siRNA were treated with olaparib for 24 h. Representative histograms indicated the percentages of colony formation and apoptotic cells (annexin V–positive cells). Experiments were conducted at least three times. Bar values are presented as mean ± SD. *P < 0.05. [file 12929_2020_699_MOESM1_ESM.pptx]
